# Ferroptosis and the tumor microenvironment

**DOI:** 10.1186/s13046-024-03235-0

**Published:** 2024-11-30

**Authors:** Kaisa Cui, Kang Wang, Zhaohui Huang

**Affiliations:** 1 Wuxi Cancer Institute, Affiliated Hospital of Jiangnan University, Huihe Road 200, Wuxi, Jiangsu 214062 China; 2 Laboratory of Cancer Epigenetics, Wuxi School of Medicine, Jiangnan University, Lihu Avenue 1800, Wuxi, Jiangsu 214122 China; 3 Department of Radiology, Affiliated Hospital of Jiangnan University, Wuxi, Jiangsu 214122 China

**Keywords:** Ferroptosis, Tumor microenvironment, Cancer-associated fibroblast, Tumor-associated macrophage, CD8^+^ T cell, Immunotherapy

## Abstract

Ferroptosis is a type of regulated cell death characterized by its non-apoptotic, iron-dependent and oxidative nature. Since its discovery in 2012, extensive research has demonstrated its pivotal roles in tumorigenesis, metastasis and cancer therapy. The tumor microenvironment (TME) is a complex ecosystem comprising cancer cells, non-cancer cells, extracellular matrix, metabolites and cytokines. Recent studies have underscored a new paradigm in which non-cancer cells in the TME, such as immune and stromal cells, also play significant roles in regulating tumor progression and therapeutic resistance typically through complicated crosstalk with cancer cells. Notably, this crosstalk in the TME were partially mediated through ferrotopsis-related mechanisms. This review provides a comprehensive and systematic summary of the current findings concerning the roles of ferroptosis in the TME and how ferroptosis-mediated TME reprogramming impacts cancer therapeutic resistance and progression. Additionally, this review outlines various ferroptosis-related therapeutic strategies aimed at targeting the TME.

## Introduction

Cell death is triggered by sensing mechanisms in cells suffering serious damage or stress, and resistance to cell death is a hallmark of cancer, highlighting the pivotal role of cell death in tumorigenesis and progression [1]. To sustain uncontrollable proliferation, cancer cells must suppress or prevent the activation of cell death pathways [1]. Inevitable variants of cellular demise are typically classified as accidental cell death, whereas cell death initiated by a genetically encoded apparatus in most contexts is referred to as regulated cell death (RCD) [2]. Ferroptosis represents a novel form of RCD driven by iron-dependent lipid peroxidation [3]. Intriguingly, tumor suppressors can execute partial tumor-suppression functions depending on ferroptosis, which seems to be an important tumor-suppressive mechanism [4]. For instance, the well-known tumor suppressor p53 sensitizes tumor cells to ferroptosis by inhibiting solute carrier family 7 member 11 (SLC7A11, also named xCT), a cystine/glutamate antiporter component [5]. In addition, other tumor suppressors such as BRCA1 associated deubiquitinase 1 (BAP1), fumarate hydratase (FH), lactamase beta (LACTB), kelch like ECH associated protein 1 (KEAP1) and lysine methyltransferase 2B (KMT2B) have been shown to exert their tumor-inhibitory functions by, at least partially, inducing ferroptosis in tumor cells [4, 6, 7]. However, cancer cells can evolve various mechanisms to evade ferroptosis [4]. Consistently, the inactivation of a series of tumor suppressors, such as p53 and BAP1, can increase SLC7A11 expression to confer ferroptosis evasion, promoting tumor growth [4]. These observations underscore the complex roles of ferroptosis in cancer therapy and progression.

In tumor tissues, cancer cells, immune cells, stromal cells and other components constitute the tumor microenvironment (TME), an ecosystem closely intricately linked to tumor initiation and progression. These various cell types can communicate with each other directly or indirectly, such as through cancer cell-secreted extracellular vesicles (EVs) that regulate non-cancer cells in the TME [8–11]. Research on the TME over the past decades has greatly challenged conclusions drawn from traditional experimental systems based solely on cancer cell models. For instance, several studies have shown that some patients have a poor prognosis despite high immune cell infiltration in the TME, suggesting that relying on a single immune evaluation may lead to misclassification of risk in certain cancer types [12–14].

Many studies have demonstrated the emerging role of cancer cell ferroptosis in tumorigenesis, metastasis and therapeutic resistance. RCD occurs not only due to microenvironmental perturbations but also because of tissue homeostasis and immune responses [2]. Notably, in the past two years, reports focusing on immune and stromal cell ferroptosis in the TME have rapidly increased. In this review, we briefly review the core pathways regulating ferroptosis, provide a comprehensive summary of ferroptosis-related crosstalk between cancer cells and non-cancer cells within the TME, and present an updated overview of ferroptosis-related molecular signatures and therapeutic strategies in the TME.

## Ferroptosis

**Fig. 1:**
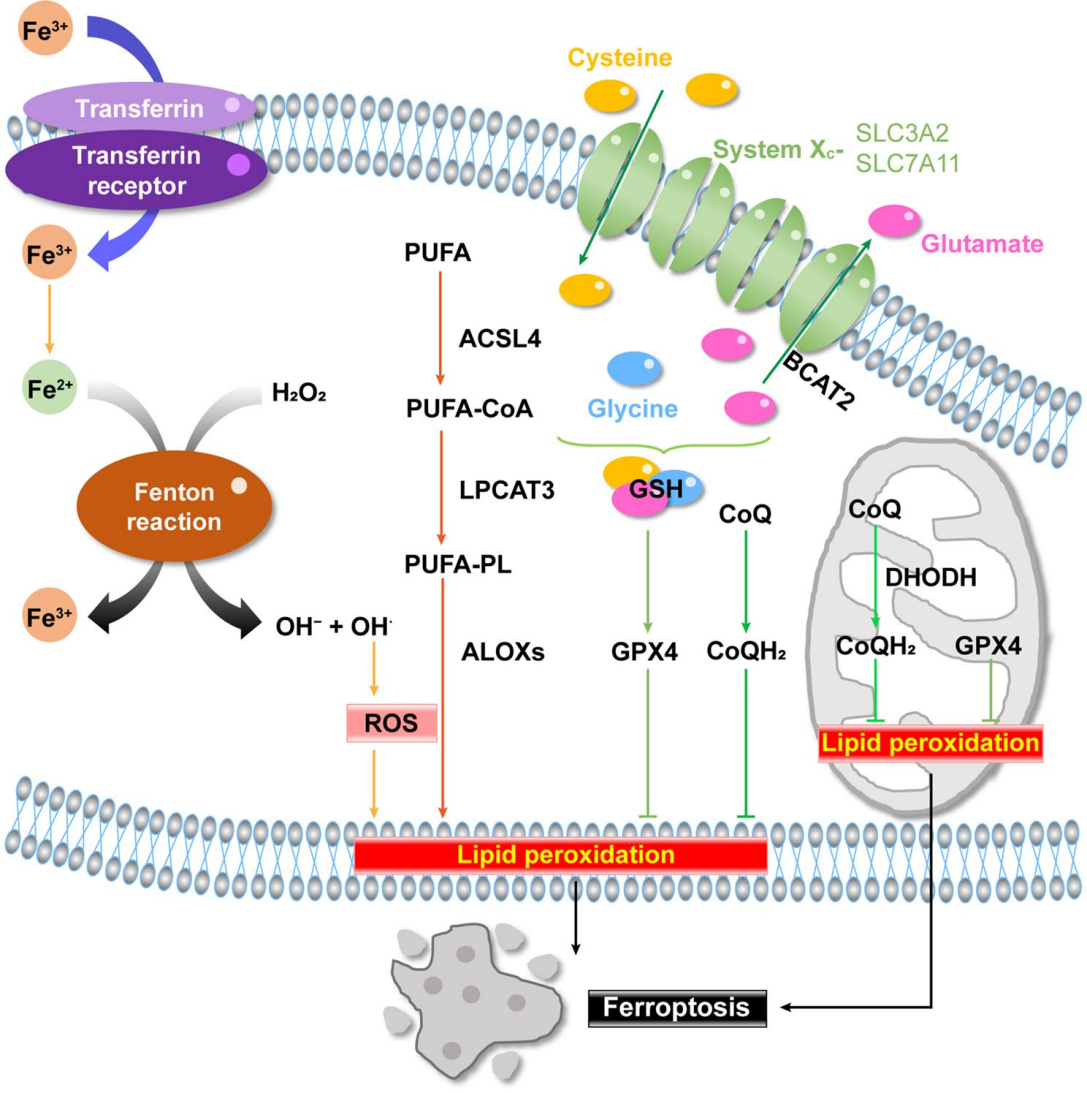
Schematic illustration of the core pathways regulating ferroptosis. The mechanisms inducing ferroptosis involve and enzymatic non-enzymatic lipid peroxidation. The mechanisms counteracting ferroptosis include the cysteine/GSH/GPX4 mechanism, FSP1 mechanism and DHODH mechanism. OH^⋅^: hydroxyl radical; ROS: reactive oxygen species; PUFA: polyunsaturated fatty acid; PL: Phospholipids; ACSL4: Acyl-CoA synthetase long chain family member 4; LPCAT3: lysophosphatidylcholine acyltransferase 3; ALOXs: arachidonate lipoxygenases; GSH: glutathione; GPX4: glutathione peroxidase 4; FSP1: ferroptosis suppressor protein 1; DHODH: dihydroorotate dehydrogenase; SLC7A11: solute carrier family 7 member 11; SLC3A2: solute carrier family 3 member 2; System X_c_^−^: Cystine-glutamate antiporter; BACT2: branched-chain amino acid aminotransferase 2; CoQ: ubiquinone-10 (also known as coenzyme Q_10)_; CoQH_2_: ubiquinol

The concept of ferroptosis was initially introduced by the Stockwell laboratory a decade ago [3]. In their pioneering work, they identified two structurally unrelated oncogenic RAS-selective lethal small molecules called erastin and RSL3 [15, 16]. Subsequent studies by the team unveiled that erastin can trigger a distinct form of cell death termed ferroptosis, which is a non-apoptotic, iron-dependent and oxidative cell death type [3]. Furthermore, they identified ferrostatin-1 as the first ferroptosis inhibitor [3]. Currently, the primary mechanism driving ferroptosis is the accumulation of peroxidized lipids within cells, and three major protective mechanisms against ferroptosis are summarized in the following sections (Fig. 1).

### Mechanisms inducing ferroptosis

#### Non-enzymatic lipid peroxidation

In addition to hydrogen, carbon, nitrogen, and oxygen, minerals constitute approximately 4% of the human body [17]. Among these minerals, 16 different types are believed to be essential, including iron, which is vitally required by the human body [17]. Iron is absorbed by the human intestinal mucosa in heme and non-heme forms, with the non-heme forms containing Fe^2+^ and Fe^3+^[17]. Once iron is bound to transferrin in the Fe^3+^ form, it is transported via the transferrin receptor [17]. Iron salts react with peroxides to generate hydroxyl radicals, which is known as the Fenton reaction (Fe^2+^ + H_2_O_2_ → Fe^3+^ + OH^−^ + OH^⋅^), driving non-enzymatic autoxidation to trigger the propagation of phospholipid peroxidation [4, 18, 19]. Both Fe^2+^ and Fe^3+^ can act as catalysts to regulate the Fenton reaction [20]. The hydroxyl radical (OH^⋅^) is the most common reactive form of reactive oxygen species (ROS) that attacks a wide range of organic molecules, initiating lipid peroxidation [21]. Therefore, this mechanism is governed by an imbalance in iron metabolism: an overload of Fe^2^^+^ within cells triggers the Fenton reaction with hydrogen peroxide, ultimately leading to ferroptosis [22].

#### Enzymatic mechanism of lipid peroxidation

Phospholipids (PLs) are fundamental components of cell membranes, with polyunsaturated fatty acids (PUFAs) in PLs serving as essential peroxidation substrates for ferroptosis [18]. ROS can react with PUFA-containing PLs, leading to the production of lipid hydroperoxides catalyzed by iron [19]. These lipid hydroperoxides can then participate in Fenton-like reactions as substrates, generating alkoxyl radicals to initiate lipid peroxidation [19]. Fenton-like reactions, involving metals such as copper, cobalt, manganese, cerium, aluminum, chromium, and ruthenium, can not only produce H_2_O_2_ but also generate persulfate and peroxymonosulfate, which further generate OH^⋅^ [23].

Acyl-CoA synthetase long chain family member 4 (ACSL4) and lysophosphatidylcholine acyltransferase 3 (LPCAT3) are the first identified pro-ferroptotic genes, and play pivotal roles in the activation and incorporation of PUFAs like arachidonic acid (AA) into membrane-localized lipids [24]. The initial study revealed that ferroptosis induction is specifically linked to ACSL4 rather than other members of the ACSL family. ACSL4 preferentially activates AA to AA-CoA, leading to the generation of phosphatidylethanolamine (PE)-AA that are subsequently esterified by LPCAT3 [4, 24]. This process ultimately triggers lipid peroxidation through arachidonate lipoxygenases (ALOXs), which represent an enzymatic reaction-driven pathway that initiates the formation of lipid hydroperoxides [4, 18]. Therefore, the hallmark of ferroptosis induction is ROS-mediated lipid peroxidation. Additionally, the excessive accumulation of oxidized PUFA-containing lipids within specific cell membranes is necessary for ferroptosis induction [18].

### Mechanisms against ferroptosis

Three major protective mechanisms against ferroptosis have been identified in cells: the cysteine/glutathione (GSH)/glutathione peroxidase 4 (GPX4) mechanism, the ferroptosis suppressor protein 1 (FSP1) mechanism, and the dihydroorotate dehydrogenase (DHODH) mechanism.

#### The cysteine/GSH/GPX4 mechanism

GSH is a tripeptide compound comprising glutamate, cysteine and glycine, and functions as an important antioxidant and free radical scavenger [22]. Cystine-glutamate antiporter (system X_c_^−^), consisting of a light (SLC7A11) and a heavy chain (solute carrier family 3 member 2, SLC3A2) subunit, enables the uptake of cystine from the extracellular environment [4, 22]. Branched-chain amino acid aminotransferase 2 (BCAT2), a key enzyme in amino acid metabolism, regulates the intracellular glutamate level [25]. Elevated extracellular glutamate levels impede intracellular cystine/cysteine uptake, triggering ferroptosis. Conversely, BCAT2 increases intracellular glutamate levels, promoting the release of glutamate and enhancing system X_c_^−^ activity to protect cancer cells from ferroptosis [25].

GPX4, a selenoprotein, plays a critical role in suppressing ferroptosis. Among the GPX family members, GPX4 is a GSH-dependent peroxidase that counteracts the oxidation of lipids in membranes [18, 26]. Depletion of GSH inactivates GPX enzymes, and GPX4 is further directly inhibited by RSL3, the second ferroptosis inducer that triggers ferroptosis independently of voltage dependent anion channel 2/3 (VDAC2/VDAC3) or system X_c_^−^ [15, 27]. Additionally, GPX4 modulates ferroptotic cell death triggered by various ferroptosis inducers, but not other cell death types induced by non-ferroptosis inducers in cancer cells [27].

#### The FSP1 mechanism

The FSP1-mediated ferroptosis suppression pathway is a parallel system that co-operates with cysteine/GSH/GPX4-mediated ferroptosis suppression. In 2019, two studies published in *Nature* identified FSP1 as a glutathione-independent ferroptosis resistance factor [28, 29]. *Ubiquinone-10 (CoQ*,* also known as coenzyme Q*_*10*_*)* plays a vital role in crucial electron transport for energy production in mitochondria [4, 28]. Ubiquinol (CoQH_2_) is the reduced form of CoQ, and FSP1 can catalyze the regeneration of CoQ to CoQH_2_, a radical-trapping antioxidant with ferroptosis- suppressive activity [28–30].

#### The DHODH mechanism

The second GPX4-independent system involves DHODH-mediated ferroptosis suppression. Pyrimidine de novo synthesis involves six chemical reactions catalyzed by three enzymes, including cytosolic dihydroorotase (CAD), mitochondrial inner membrane-anchored DHODH, and cytosolic uridine 5′-monophosphate synthase (UMPS) [31]. Administration of dihydroorotate or orotate, the substrate or product of DHODH, respectively, can attenuate or potentiate the ferroptosis induced by GPX4 inhibition, especially evident in tumor cells expressing low levels of GPX4 [30]. GPX4 is present in various cell compartments, including mitochondria, cytosol, and nucleus [4]. Impressively, DHODH operates in parallel with mitochondrial GPX4 to inhibit ferroptosis in the mitochondrial inner membrane by converting CoQ to CoQH_2_, without relying on cytosolic GPX4 or the FSP1 pathway [30]. Furthermore, glutamate oxaloacetate transaminase 1 (GOT1) and mitochondrial GOT2 synthesize aspartate, a direct precursor of CAD [31]. A recent study revealed that the cytosolic proteins GOT1, CAD and UMPS form a multi-enzyme complex called “pyrimidinosome”, which links to the mitochondrial inner membrane-anchored DHODH and thereby regulates ferroptosis [31]. These findings unveil a DHODH-mediated mechanism against ferroptosis, presenting a promising avenue for cancer therapy targeting ferroptosis.

## Ferroptosis-related roles in the TME

The TME comprises various components, encompassing not only tumor cells but also stromal and immune cells. Each cell type in the TME can be further characterized by multiple markers to exhibit a range of phenotypes [32]. These components organize in spatially structured arrangements, showing microenvironmental niches, nutrient gradients, and cell‒cell interaction phenotypes [32]. Multiple non-tumor cell types in the TME, such as cancer-associated fibroblasts (CAFs) and tumor-associated macrophages (TAMs), drive a chronic inflammatory, immunosuppressive, and pro-angiogenic intratumoral environment that supports tumor growth and metastasis [33]. Ferroptotic cell-released metabolites or damage-associated molecular patterns in the TME can induce immune responses of tumor-infiltrating immune cells, suggesting ferroptosis as an immunogenic cell death.

### Ferroptosis and CAFs in the TME

**Fig. 2:**
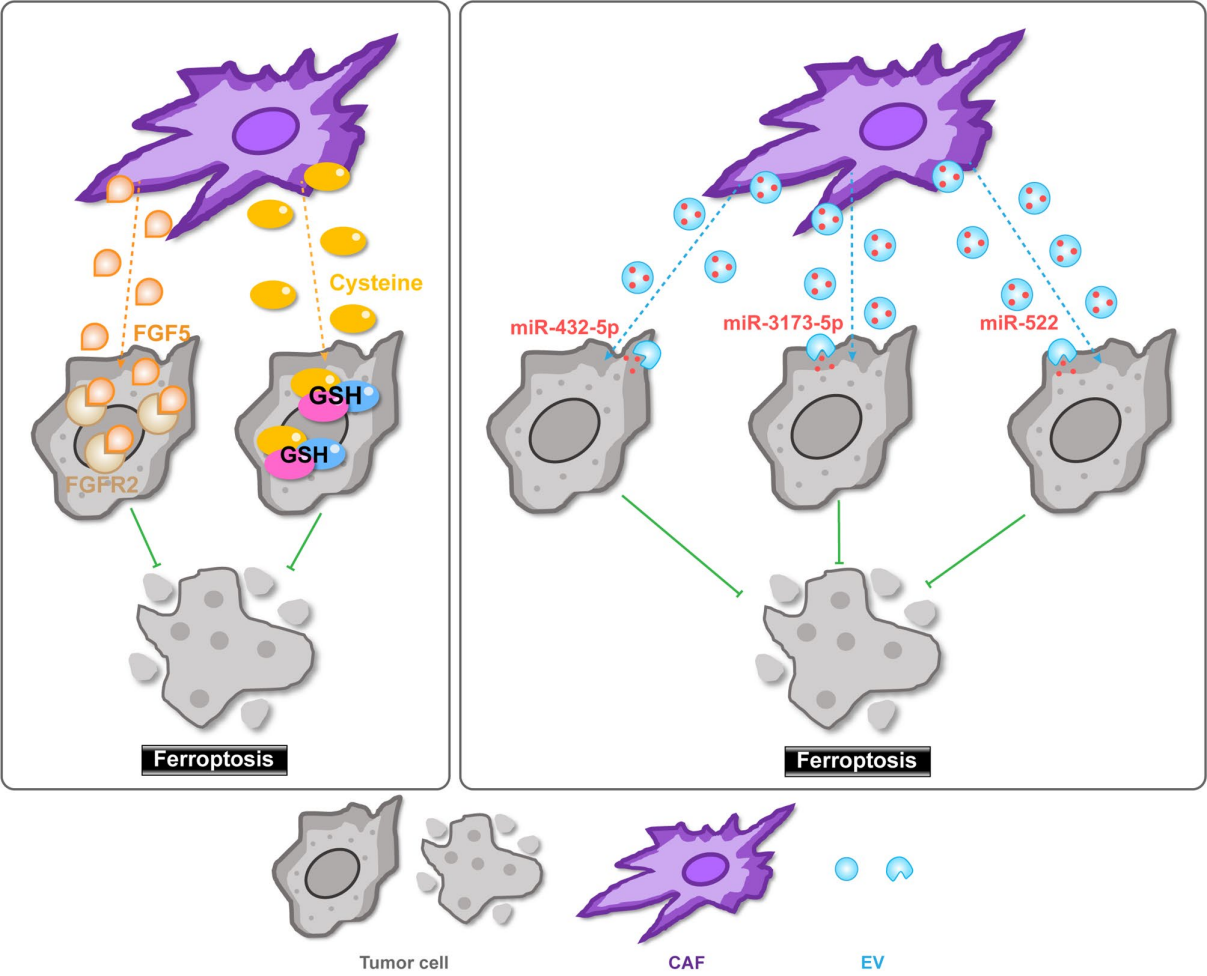
Schematic illustration of ferroptosis and interactions with CAFs in the TME. CAFs can regulate tumor cell ferroptosis within the TME by secreting proteins, cysteine or EVs. Conversely, tumor cell ferroptosis also influences CAF functions. CAF: cancer-associated fibroblast; TME: tumor microenvironment; EV: extracellular vesicle. ACSL4: Acyl-CoA synthetase long chain family member 4; GSH: glutathione; ROS: reactive oxygen species; FGF5: fibroblast growth factor 5; FGFR2: fibroblast growth factor receptor 2

Mesenchymal stromal cell-derived stromal cells are vital components of the TME and key regulators of tumor progression [34]. CAFs are activated fibroblastic cells with a spindle-like morphology in the TME, and phenotypes distinct from those of quiescent fibroblasts in normal tissues [35]. In certain cancer types, CAFs are the most prominent stromal cell type, and their abundance is often associated with poor prognosis [36]. CAFs were previously considered a relatively uniform population of matrix-producing cells. However, recent single-cell RNA sequencing (scRNA-Seq) studies have unveiled diverse CAF phenotypes and subpopulations [37]. CAFs can regulate tumor cell ferroptosis within the TME (Fig. 2). For example, in nasopharyngeal carcinoma, CAFs secrete high levels of *fibroblast growth factor 5 (FGF5)*, which binds to *fibroblast growth factor receptor 2 (FGFR2)* in tumor cells, hence inhibiting cisplatin-induced ferroptosis [38]. In addition, through the TGF-β/SMAD3/ATF4 signaling axis, CAFs secrete cysteine, enhancing the extracellular supply of cysteine to support GSH synthesis, thereby inducing tumor cell ferroptosis resistance in pancreatic ductal adenocarcinoma [39].

Conversely, tumor cell ferroptosis also influences CAF function (Fig. 2). Anoctamin 1 (ANO1) overexpression in gastrointestinal cancer cells is associated with CAF abundance and adverse immunotherapeutic outcomes. ANO1 suppresses tumor cell ferroptosis by regulating the PI3K‒Akt signaling axis, promoting the production and secretion of TGF-β by cancer cells to recruit CAFs, and conferring immunotherapeutic resistance [40].

EVs, including exosomes and microvesicles, are cell-derived membranous structures that play crucial roles in both the local and systemic effects of tumor progression [10, 41, 42]. Locally, EVs mediate intercellular communication within the TME, transferring proteins, lipids and nucleic acids to recipient cells, such as cancer cells, CAFs (Fig. 2) and macrophages [10]. For example, upon uptake by cancer cells, CAF-secreted exosomal miR-3173-5p sponges ACSL4 and thereby suppresses ferroptosis, inducing acquired gemcitabine resistance in pancreatic cancer [43]. Chemotherapy induces CAFs to secrete exosomal miR-522, which suppresses ALOX15 expression and lipid ROS accumulation in gastric cancer cells, dampening cancer cell ferroptosis and chemosensitivity [44]. Additionally, CAF-derived exosomal miR-432-5p targets CHAC1, reducing GSH consumption, inhibiting ferroptosis, and conferring chemoresistance to docetaxel in prostate cancer cells [45].

### Ferroptosis and myeloid cells in the TME

Myeloid cells are a heterogeneous group of innate immune cells and are the most abundant immune cell type in the TME, exerting both immunosuppressive and immunostimulatory effects. Myeloid cells include mainly macrophages, neutrophils and dendritic cells (DCs) [46]. DCs are professional antigen-presenting cells capable of stimulating naïve T cells. Most DC-related studies focused on ferroptosis-mediated TME reprogramming in cancer therapy by inducing DC maturation or enhancing DC antigen presentation, as discussed in the related sections.

#### TAMs

Macrophages are not a uniform population with a defined phenotype but rather a diverse collection of cell types with varying functions under different physiological and pathological conditions. However, within the TME, tumor cells can skew this proclivity to stimulate tumor proliferation, angiogenesis, and metastasis [47]. Macrophages in tumor tissues, known as TAMs, play a critical role in inducing an inhibitory TME. TAMs are generally polarized into two categories, M1-like and M2-like macrophages, which exhibit pro- and anti-inflammatory properties, respectively. M1-like macrophages produce angiostatic and pro-inflammatory factors that are associated with anti-tumor immunity, whereas M2-like TAMs secrete tissue-remodeling and pro-angiogenic factors to promote tumorigenesis and tumor progression [48, 49].

Studies have revealed the significance of ferroptosis in TAMs. In 2017, Wang et al. revealed that iron overload induces Slc7a11-mediated ferroptosis in murine hepatocytes and macrophages, marking the first observation of ferroptosis in macrophages [50]. The phagocytosis of ferroptotic cells by macrophages is important for eliminating dying tumor cells during cancer therapy. Luo et al. identified a novel pathway involving the clearance of ferroptotic cells by macrophages through a key “eat-me” oxidized phospholipid on the ferroptotic tumor cell surface, 1-steaoryl-2-15-HpETE-sn-glycero-3-phosphatidylethanolamine (SAPE-OOH) [51]. Moreover, they revealed that phospholipid peroxidation in TAMs impairs their phagocytic towards ferroptotic tumor cells, thus contributing to resistance to ferroptosis therapy [52]. Mechanistically, SAPE-OOH was identified as a key factor to mediate these effects by promoting ubiquitin degradation of TLR2 [52].

**Fig. 3:**
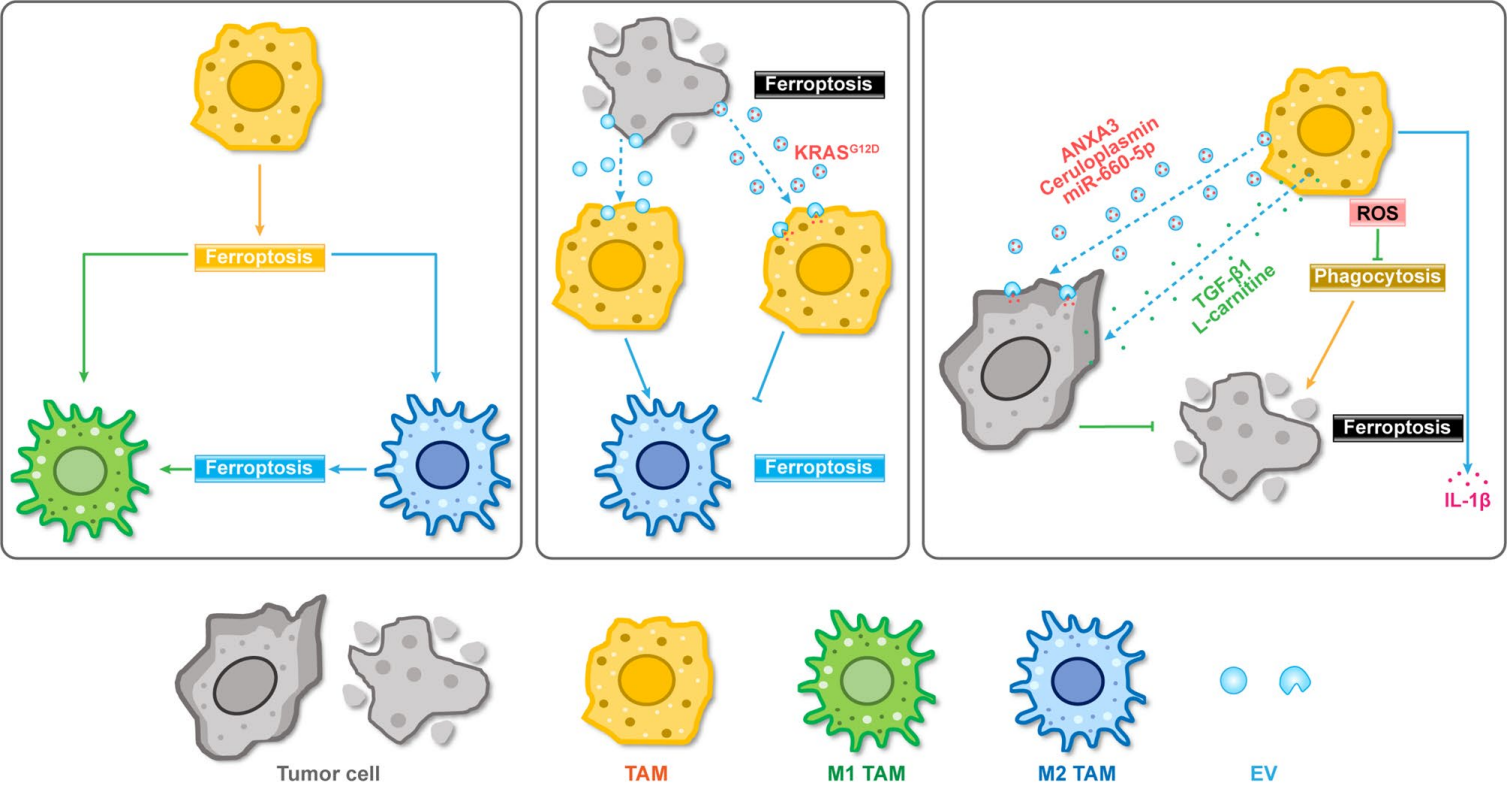
Schematic illustration of ferroptosis and interactions with TAMs in the TME. Ferroptosis and related inducers/inhibitors can mediate TAM reprogramming. TAMs can regulate cancer cell ferroptosis via paracrine effects. EV: extracellular vesicle; ROS: reactive oxygen species; TAMs: tumor-associated macrophages

Recent studies have shown multiple correlations between ferroptosis and macrophage reprogramming (Fig. 3). For example, multiple ferroptosis activators can induce HMGB1 release in the TME, which mediates immune response in macrophages by upregulating TNFα [53]. In addition, microparticles released by irradiated tumor cells can reprogram M2-like TAMs into M1-like macrophages, inducing immunogenic death primarily through ferroptosis [54]. Erastin- and ACSL4-induced tumor cell ferroptosis can promote M1 macrophage polarization, inhibiting the growth of nasopharyngeal carcinoma [55]. Short-term acidosis can induce M1 macrophage polarization to promote tumor cell ferroptosis via the ZFAND5/SLC3A2 signaling axis in breast cancer [56]. APOC1 inhibition can promote M2 TAMs to repolarize into M1 macrophages through the ferroptosis pathway in HCC [57]. In lung cancer, dihydroartemisinin induces ferroptosis in TAMs and promotes their phenotypic shift toward M1 macrophages by activating NF-κB signaling [58]. Targeting xCT in TAMs suppresses tumorigenesis and metastasis by inducing ferroptois within TAMs, inhibiting macrophages recruitment and M2 polarizaion [59, 60]. These data highlight the key role of ferroptosis in restoring the immune acitivities of TAMs.

In addition to the aforementioned immunostimulatory effects of ferroptosis on macrophages, some studies reported the ferroptosis-related immunosuppressive effects on TAMs (Fig. 3). For instance, when aggregated TAMs encounter ferroptotic liver tumor cells, these macrophages can secrete pro-inflammatory IL-1β to promote aggressive tumor growth and metastasis [61]. Hepatocellular ferroptosis promotes the activation of STING signaling in macrophages, which in turn facilitates hepatocellular carcinoma (HCC) progression [62]. In addition, ferritin light chain (FTL) promotes M2 polarization of TAMs by facilitating the ferroptosis pathway through the inhibition of iPLA2β in glioma [63]. Interestingly, a low concentration of erastin can effectively induce M2 macrophage polarization through a ferroptosis-independent mechanism, promoting the metastasis of ferroptosis-resistant ovarian cancer cells [64].

TAMs can regulate cancer cell ferroptosis through paracrine signaling (Fig. 3). For instance, TAM-derived TGF-β1 induces ferroptosis resistance in triple-negative breast cancer (TNBC) cells, promoting chemoresistance and cancer progression [65]. CPT1A, a fatty acid oxidation rate-limiting enzyme, can collaborate with TAM-derived L-carnitine to induce ferroptosis resistance in lung cancer cells [66]. Additionally, TAMs can transfer bioactive molecules via EVs to regulate cancer cell functions. For instance, TAM-derived EVs can deliver ANXA3, ceruloplasmin mRNA, and miR-660-5p to cancer cells, regulating their ferroptosis [67–69]. Conversely, tumor cells can also secrete EVs to regulate the phenotypes of TAMs [8]. Ferroptotic breast cancer cell-derived exosomes inhibit M2 macrophage polarization, thereby suppressing breast cancer metastasis [70]. Oxidative stress induces pancreatic ductal adenocarcinoma cells to succumb to autophagy-dependent ferroptosis, releasing KRAS^G12D^ protein-rich exosomes, which induce TAM M2 polarization via STAT3-dependent fatty acid oxidation, promoting tumor growth [71]. Overall, EVs are important factors and mechanisms that mediate ferroptosis-related communication between tumor cells and macrophages.

#### Neutrophils

Neutrophils, previously known as polymorphonuclear leukocytes, originate from haematopoietic progenitor stem cells in the bone marrow [72]. These cells are abundant in human blood and are generally characterized by a short lifespan, terminal differentiation, and nonproliferative nature [72]. Hence, the functional diversity of neutrophils has not been extensively explored compared with that of other myeloid cell types [72]. The potential procarcinogenic effects of neutrophils were first reported by Knaapen et al. in 1999 [73]. They reported that neutrophils release ROS, leading to oxidative DNA damage in epithelial cells, suggesting potential connections between neutrophils and ferroptosis [73].

According to the scRNA-Seq data of pediatric hepatoblastoma, intratumoral neutrophils consist of heterogeneous functional populations at different development stages. Specifically, terminally differentiated neutrophils with active ferroptosis predominate in tumor tissues, and the CXCR4^+^ neutrophil subpopulation shows a greater ferroptosis tendency and immunosuppressive phenotype than other subpopulations [74]. However, on the base of the scRNA-Seq data of murine mammary tumor models, *aconitate decarboxylase 1* (Acod1) emerges as the most upregulated metabolic enzyme in mouse *tumor-associated neutrophils (TANs)* [75]. An Acod1^+^ TAN subset, which defends against ferroptosis and promotes cancer metastasis via an Acod1-dependent immunometabolism switch, has also been identified in human breast tumors [75]. Therefore, ferroptosis can play contrasting roles in neutrophil-related tumor-promoting and immunosuppressive functions.

Approximately half of all cancer-related deaths worldwide can be attributed to a wasting condition known as “cachexia”, characterized by ongoing loss of adipose and muscle tissues [76]. Recent research has shown that in the model of lung cancer cachexia, tissue-infiltrating neutrophils increase and secrete Lipocalin-2 (LCN2), inducing ferroptosis that causes tissue wasting [76]. Tissue death in cancers, generally referred to as “tumor necrosis”, is a common feature and a poor prognostic indicator in human advanced cancers [77]. Interestingly, neutrophils have been reported to induce glioblastoma ferroptosis and thereby promote tumor necrosis by transferring myeloperoxidase-containing granules into cancer cells, revealing a new pro-tumorigenic role of ferroptosis [77].

Immunosuppressive neutrophils generally exhibit a more immature phenotype, although mature immunosuppressive neutrophils have been described in the bloodstream. Therefore, the term ‘myeloid-derived suppressor cells’ (MDSCs) is also used to describe *TANs* [72]. Pathologically activated neutrophils (PMNs), termed PMN-MDSCs, are major negative regulators of anti-tumor immunity [78]. The ferroptosis of PMN-MDSCs induces the release of oxygenated lipids, inhibiting T-cell activity and fostering an immunosuppressive microenvironment [78]. Additionally, although GPX4 loss-induced ferroptosis may not directly inhibit cancer cell tumorigenesis, ferroptosis can induce immunosuppression by promoting MDSC recruitment [79]. Consequently, targeting neutrophils might offer potential benefits to patients with cancer-related histopathological issues via the modulation of ferroptosis.

### Ferroptosis and T lymphocytes in the TME

**Fig. 4:**
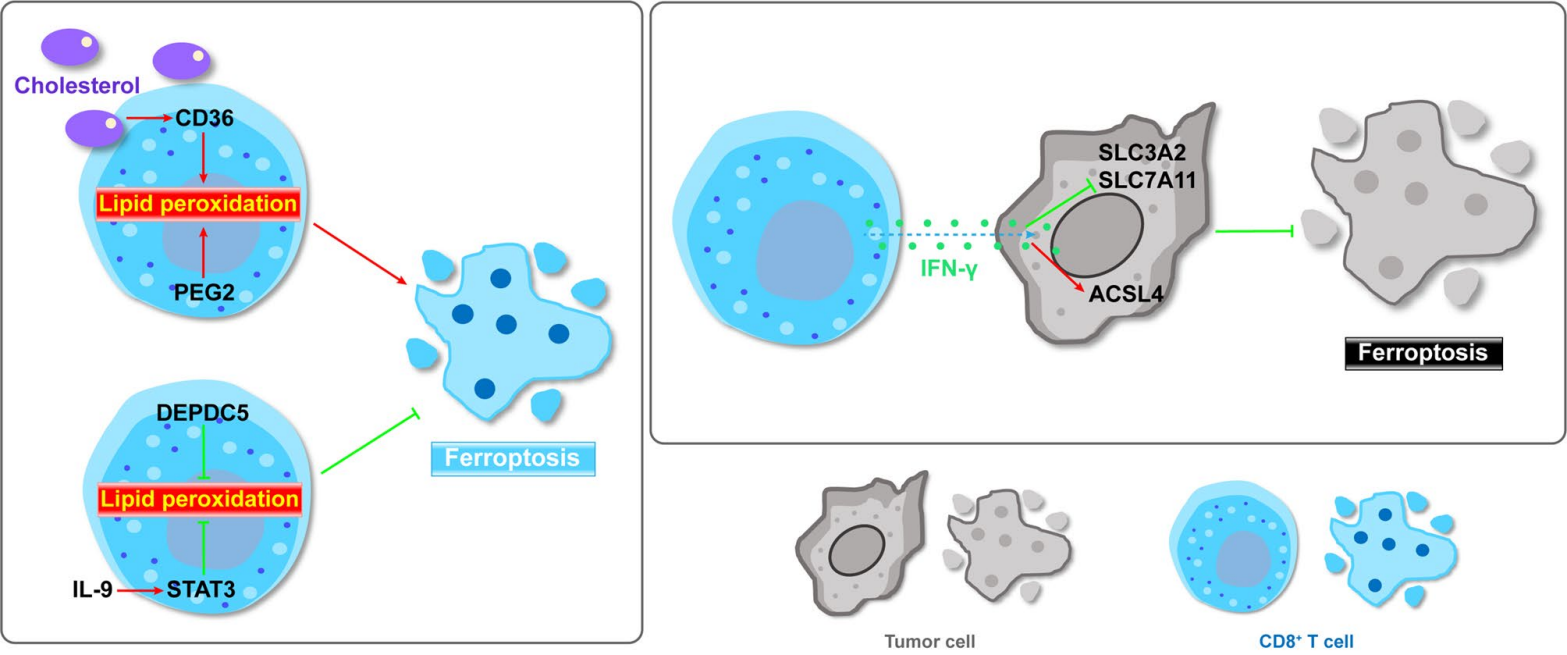
Schematic illustration of ferroptosis and interactions with CD8^+^ T cells in the TME. High cholesterol levels in the TME can induce ferroptosis in CD8^+^ T cells, and tumor cells can counteract anti-tumor CD8^+^ T cells by competing for cystine uptake. Anti-tumor CD8^+^ T cells, in turn, can release IFN-γ to induce ferroptosis in tumor cells. IL-9: Interleukin 9; ACSL4: Acyl-CoA synthetase long chain family member 4; IFN-γ: secreting interferon gamma; DEPDC5: DEP domain containing 5; PGE2: prostaglandin E2; SLC7A11: solute carrier family 7 member 11; SLC3A2: solute carrier family 3 member 2; GSH: glutathione

T lymphocytes, originating from bone marrow progenitors and undergoing differentiation and maturation in the thymus, play a central role in cellular immunotherapy [80]. The primary distinction among T cells is that of CD8^+^ and CD4^+^ T cells [81]. Current research on T cells and ferroptosis in human cancers predominantly focuses on CD8^+^ T cells (Fig. 4).

Initially, CD8^+^ T cells were considered uniformly cytotoxic cells responsible for eliminating infected or tumorigenic cells by secreting interferon gamma (IFN-γ) and cytolytic granules [82]. IFN-γ plays essential roles in homeostasis, immune responses and tumor immunosurveillance [83]. The presence of tumor-infiltrating lymphocytes (TILs), particularly CD8^+^ T cells, is associated with favorable prognosis in various human cancers. Advancements in technologies, such as cytometry by time of flight (CyTOF) and scRNA-seq, have allowed the CD8^+^ T cells to be subdivided substantially [81]. Novel CD8^+^ T cell subpopulations with an immunosuppressive phenotype have been identified within tumors and are often associated with poor survival [84–86]. These CD8^+^ T cells always exhibit exhausted signatures, indicating a dysfunctional state.

CD8^+^ T cells have developed mechanisms to evade ferroptosis in the TME. Previous studies have shown that cholesterol in the TME can induce CD8^+^ T cell functional exhaustion, and that inhibiting cholesterol metabolism in CD8^+^ T cells can restore their anti-tumor activities, presenting a novel strategy to enhance T cell-based cancer immunotherapy [87]. Further investigations revealed that cholesterol in the TME induces CD36 overexpression in tumor-infiltrating CD8^+^ T cells [88]. CD36 facilitates the uptake of fatty acids by CD8^+^ T cells in the TME, inducing lipid peroxidation and ferroptosis, thus impairing the anti-tumor capabilities of CD8^+^ T cells [88]. CD8^+^ T cells can be classified into various subpopulations such as Tc1, Tc2, Tc9, Tc17, and Tc22 [89]. Among these, CD8^+^ Tc9 cells are characterized by interleukin 9 (IL-9) production but limited IFN-γ and cytotoxic functions [89]. Adoptively transferred tumor-specific Tc9 cells exhibit a robust anti-tumor response against advanced tumors [90]. Similarly, cholesterol has also been shown to inhibit the differentiation and anti-tumor activity of Tc9 CD8^+^ T cells [91]. Tc9 cells possess unique lipid metabolic programs and Tc9 cell-secreted IL-9 activates STAT3 and increases fatty acid oxidation and mitochondrial activity, rendering Tc9 cells with decreased lipid peroxidation and resistant to ferroptosis [82].

In a recent study, Li et al. reported that DEP domain containing 5 (DEPDC5) expression is positively correlated with tumor-infiltrating CD8^+^ T cells and favorable cancer patient survival [92]. Mechanistically, DEPDC5 protects CD8^+^ T cells from ferroptosis by reducing ROS production and lipid peroxidation via the inhibition of mTORC1-mediated purine catabolism [92]. The expansion of antigen-experienced CD8^+^ T cells is important for TIL-based adoptive cell therapy (ACT) in cancer patients. Prostaglandin E2 (PGE2), a negative regulator of the immune response, can downregulate the IL-2Rγc chain and impair the assembly of IL-2Rβ–IL2Rγc membrane dimers, leading to oxidative stress and ferroptosis in tumor-reactive TILs [93]. During TIL expansion for ACT, the signaling of PGE2 to EP2 and EP4 is inhibited, increasing IL-2 sensing and enhancing the proliferation of tumor-reactive TILs [93]. Overall, CD8^+^ T cells employ multiple mechanisms to evade ferroptosis in the TME, offering insights for the development of T cell-based cancer immunotherapy.

On the other hand, tumor cells have developed various strategies to counteract anti-tumor CD8^+^ T cells and evade their cytotoxic effects. IFN-γ plays a pivotal role in this dynamic interplay. SLC7A11, a functional subunit of system X_c_^−^, is upregulated in tumors and aids tumor cells to outcompete CD8^+^ T cells for cystine uptake, ultimately inducing CD8^+^ T-cell exhaustion and ferroptosis [94]. Conversely, ferroptosis can function as a mechanism for anti-tumor CD8^+^ T cell-mediated tumor cell killing, with IFN-γ playing a crucial role in regulating this communication. Immunotherapy-activated CD8^+^ T cells can release IFN-γ to downregulate the system X_c_^−^ functional subunits SLC3A2 and SLC7A11, impeding tumor cells from acquiring cystine and thus leading to lipid peroxidation and ferroptosis in cancer cells [95]. Similarly, another study reported that CD8^+^ T cell-secreted IFN-γ suppresses SLC7A11, promoting cancer cell ferroptosis and thereby sensitizing cancer cells to radiotherapy [96]. Moreover, CD8^+^ T cell-derived IFN-γ and arachidonic acid from the TME induce cancer cell ferroptosis by stimulating ACSL4 expression. Targeting arachidonic acid metabolism sensitizes cancer cells to immune checkpoint blockade therapy [97]. In addition, the loss of methionine adenosyltransferase 1 A (MAT1A) in HCC induces ferroptosis suppression and immune escape. Restoring MAT1A expression increases S-adenosylmethionine levels, boosting CD8^+^ T cell activity and promoting tumor cell ferroptosis directly or indirectly [98].

Regulatory T cells (Tregs), constituting 5–7% of CD4^+^ T cells, predominantly express FOXP3, repressing anti-tumor immunity and promoting tumor growth [99]. GPX4 shields Treg cells from lipid peroxidation and ferroptosis in the TME [100]. Treg-specific deletion of GPX4 results in excessive accumulation of lipid peroxides and ferroptosis in Tregs, repressing tumor growth and potentiating anti-tumor immunity concomitantly [100]. In addition, a recent study revealed that LINC00942 inhibits HCC ferroptosis and induces Treg immunosuppression by recruiting IGF2BP3 to stabilize SLC7A11 mRNA [101].

### Ferroptosis and natural killer (NK) cells in the TME

NK cells are unique innate lymphoid cells with intrinsic abilities to combat cancer and viral infections. However, in cancer patients, NK cells typically display a dysfunctional phenotype and reduced cytotoxic function, making the restoration or enhancement of NK-mediated anti-cancer activities a promising cancer treatment strategy [102, 103]. Tumor cells and their partners can impact NK cell survival though ferroptosis. For instance, gastric cancer cells release L-kynurenine, which induces NK cell ferroptosis in an *aryl hydrocarbon receptor*-independent manner [104]. In addition to cancer cells, CAFs can also regulate NK cell ferroptosis. Yao et al. reported that CAFs facilitate iron export into the gastric cancer TME, increasing the labile iron pool within NK cells and ultimately inducing ferroptosis in NK cells [105]. In addition, CAF-derived follistatin like protein 1 (FSTL1) increases NCOA4 expression in NK cells and then promotes NCOA4-mediated ferritinophagy and ferroptosis [105].

## Ferroptosis molecular signatures and the TME

Given the important role of ferroptosis in cancer, can ferroptosis levels serve as a guide for cancer treatment? However, defining ferroptosis levels directly via related indices, such as *malonaldehyde* or lipid ROS levels, in large numbers of patient samples or single-cell examinations remains challenging [106]. Therefore, multiple studies have identified gene expression signatures that indirectly mirror ferroptosis levels.

In 2020, Zhou et al. developed FerrDb, the first comprehensive ferroptosis database [107], which was updated in 2023 [108]. This dataset comprises a range of ferroptosis regulators manually curated from relevant literature. Leveraging FerrDb, He et al. devised a ferroptosis score (FPS) model to assess the ferroptosis status in melanoma patients [109]. A high FPS is correlated with favorable patient outcomes and immune cell infiltration. Furthermore, the FPS model is robust and capable of predicting immunotherapy response via bulk, single-cell transcriptome, and proteomic data. Intriguingly, Meng et al. reported that cancer cell sensitivities to drugs targeting epigenetic regulators, such as bromodomain and extra-terminal domain (BET) inhibitors, were strongly associated with elevated FPS [110]. BET inhibitors enhance GPX4 inhibition-induced ferroptosis in melanoma [110]. Similarly, Tang et al. calculated the ferroptosis score based on 30 ferroptosis regulators in human cancers sourced from FerrDb, and confirmed that show that the ferroptosis score is a valuable prognostic factor to evaluate immunotherapy effects [111]. These findings demonstrated the efficacy of the ferroptosis score in predicting ferroptotic cell death in tumor samples, positioning it as an independent prognostic indicator and a predictor of cancer immunotherapy outcomes.

Moreover, Han et al. utilized FerrDb to devise a seven-gene ferroptosis-related prognostic model for glioma, offering potential applications in predicting glioma prognosis and immune status [112]. In addition, Chung et al. developed a ferroptosis signature linked to inflammation/immune activation in head and neck squamous cell carcinoma [113]. Nevertheless, the aforementioned ferroptosis molecular signatures were constructed using ferroptosis regulators without differentiating between driving and suppressive regulators. We previously established a novel relative ferroptosis level (RFL) model based on ferroptosis-driving and ferroptosis-suppressive regulators across human cancers [106]. The RFL model was successfully validated in ferroptotic drug treatment datasets, unveiling several previously unreported RFL-related candidate genes associated with erastin-induced ferroptosis [106]. Moreover, a high RFL is associated with favorable patient survival, high immune cell infiltration in the TME, and improved therapeutic efficacy [106]. Collectively, these ferroptosis molecular signatures exhibit robust correlations with immune cell levels in the TME and with therapeutic responses. In addition, these related studies provide valuable resources for accurately predicting clinical outcomes and identifying new ferroptosis-related genes and inhibitors.

## Ferroptosis-related therapeutic strategies in the TME

### Chemotherapy and molecular inhibitor-induced ferroptosis in the TME

Traditional chemotherapeutic drugs, when systemically administered, disrupt the growth and division of cancer cells, constituting the cornerstone of cancer treatment [114]. However, tumor cells often evade cell death like ferroptosis, resulting in treatment resistance. For example, the alkylating compound cisplatin disrupts DNA replication and repair, ultimately inducing cell death [114]. Cancer cells can develop resistance to cisplatin treatment through dysregulated ferroptosis signaling within the TME, proposing that inducing ferroptosis could be a potential strategy to sensitize cancer cells to cisplatin [115]. Eicosapentaenoic acid treatment significantly decreases the expression of GPX4, enhancing cisplatin-induced ferroptosis in osteosarcoma [115]. In addition, eicosapentaenoic acid can increase the number of tumor-infiltrating CD8^+^ T cells and reverse cisplatin-induced PD-L1 upregulation in osteosarcoma cells [115]. Furthermore, cisplatin induces ferroptosis in lung cancer cells and promotes N1 neutrophil polarization to increase T cell infiltration and Th1 differentiation, thereby transforming “cold” tumors into “hot” tumors [116]. Moreover, cisplatin or paclitaxel promotes exosomal miR-522 secretion from CAFs, thus regulating ferroptosis and the increased chemosensitivity of gastric cancer cells [44]. Hence, chemotherapy-induced tumor cell ferroptosis and interactions with immune cells within the TME represent potential targets for combating cisplatin chemoresistance (Table 1).

**Table 1:** Ferroptosis-related therapeutic strategies in the TME

Drugs	Effects	Cancer types	References
Eicosapentaenoic acid	Enhances cisplatin-induced ferroptosis Increases the number of tumor-infiltrating CD8^+^ T cells Reverses cisplatin-induced PD-L1 upregulation	Osteosarcoma	[115]
Cisplatin	Induces ferroptosis Promotes N1 neutrophil polarization Increase T cell infiltration and Th1 differentiation	Lung cancer	[116]
Cisplatin and Paclitaxel	Promotes exosomal miR-522 secretion from CAFs, inhibiting tumor ferroptosis	Gastric cancer	[44]
KH3 GSK3326595 TrxR EC359 iFSP1 ICG001 Dual-targeting PI3K and HDAC inhibitor	Promotes ferroptosis Enhances the efficacy of ICI treatment	HCC TNBC Head and neck cancer Ovarian cancer HCC Melanoma CRC	[117] [118] [119] [121] [122] [123] [124]
Erastin	Induces ferroptosis Reprograms TAMs Enhances the efficacy of ICI treatment	Lung cancer HCC	[59, 60]
N6F11	Induces ferroptosis Initiates anti-tumor immunity Enhances the efficacy of ICI treatment	Pancreatic cancer	[125]
Oxaliplatin-artesunate	Induces ferroptosis Boosts tumor immunogenicity	Pan-cancer	[126]
BIBR1532	Enhances IR-induced ferroptosis Initiates anti-tumor immunity Enhances the efficacy of ICI treatment	NSCLC	[132]
RT-MPs	Induces ferroptosis Repolarizes M2-TAMs to M1 macrophages Reverses the tumor-inhibitory TME	Lung cancer	[54]
RC@RMPs	Induces ferroptosis Remodels the TME	Prostate cancer	[133]
SPH_GA_ TPL@TFBF E. coli@Cu_2_O	Induces ferroptosis Remodels the TME Enhances the efficacy of ICI treatment	Breast cancer Melanoma CRC	[138] [139] [140]
MnMoOx NPs CMS PDPCA@ATRA	Induces ferroptosis Remodels the TME Enhances anti-tumor immunity by depleting GSH Enhances the efficacy of ICI treatment	CRC TNBC Lung cancer	[141] [142] [143]
Fe-MOF-RP TEP-FFG-CRApY IFNγ/uMn-LDHs MMP NDs AgFeS2 Fe_3_O_4_@GOx@Tuftsin@Lipids	Induces ferroptosis Enhances anti-tumor immunity	Melanoma Prostate cancer Breast cancer Pan-cancer TNBC Lung metastases	[144] [145] [146] [147] [148] [149]

HCC: hepatocellular carcinoma; TNBC: triple-negative breast cancer; NSCLC: non-small cell lung cancer; CRC: colorectal cancer; CAFs: cancer-associated fibroblasts; TAMs: tumor-associated macrophages; ICI: immune checkpoint inhibitor; IR: ionizing radiation; TME: tumor microenvironment; GSH: glutathione

Apart from classical chemotherapeutic agents, several inhibitors have been reported to influence ferroptosis. For example, an allosteric inhibitor *(KH3)* of phosphoglycerate mutase 1 (PGAM1) promotes HCC ferroptosis, increases the number of tumor-infiltrating CD8^+^ T cells, and enhances the efficacy of anti-PD-1 immunotherapy in HCC [117]. Protein arginine methyltransferase 5 (PRMT5), an important arginine methyltransferase linked to tumor initiation and progression, has been reported to promote ferroptosis resistance in TNBC while impairing ferroptosis resistance in other breast cancer subtypes [118]. Moreover, PRMT5 inhibitors *(such as GSK3326595)* can reverse ferroptosis resistance and enhance immunotherapy efficiency in TNBC [118]. Thioredoxin reductase (TrxR), a cellular defense system against oxidative damage and apoptosis, plays a crucial role in ferroptosis [119]. The TrxR inhibitor auranofin can promote ferroptosis and restore sensitivity to anti-PD-1 immunotherapy in head and neck cancer patients with elevated TrxR expression [119]. EC359, a first-in-class leukemia inhibitory factor/ receptor small-molecule inhibitor, exhibits anti-tumor efficacy in TNBC [120]. A recent study indicated that EC359 treatment promotes ferroptosis in ovarian cancer cells, enhancing tumor immunogenicity by fostering robust leukocyte infiltration and M1 macrophage polarization [121]. Moreover, the FSP1 inhibitor iFSP1 effectively triggers tumor cell ferroptosis, leading to increased immune cell infiltration and improved immune checkpoint blockade efficacy in HCC [122].

Wnt/β-catenin signaling, which is critical in tumorigenesis, tumor progression, and therapeutic resistance, has also been linked to ferroptosis. For instance, the β-catenin inhibitor ICG001 can potentiate melanoma cell ferroptosis to increase the efficacy of PD-1 blockade [123]. Additionally, a dual-targeting PI3K and HDAC inhibitor developed by Fan and colleagues induces tumor cell ferroptosis and a pro-inflammatory TME, potentiating anti-PD1 therapy [124].

The typical ferroptosis inducer erastin is a small molecule compound known for its ability to target and kill cancer cells through multiple mechanisms. In lung cancer and HCC, erastin has been shown to increase the sensitivity of cancer cells to anti-PD-L1 by inhibiting xCT in TAMs and then reprogramming TAMs [59, 60]. The compound N6F11 can trigger tumor cell ferroptosis by ubiquitinating GPX4, without affecting the degradation of GPX4 in immune cells like T cells, NK cells, and neutrophil cells [125]. N6F11 not only initiates anti-tumor immunity but also sensitizes cancer cells to immune checkpoint blockade [125]. In addition, Fan et al. discovered a novel form of ferroptosis inducer, oxaliplatin-artesunate, which induces ferroptosis by inhibiting the GSH-mediated ferroptosis defense pathway, enhancing the iron-dependent Fenton reaction, and causing mitochondrial lipid peroxidation [126]. Oxaliplatin-artesunate also boosts tumor immunogenicity by transforming the TME from an immunosuppressive state to an immunosensitive state [126].

### Radiotherapy-induced ferroptosis in the TME

Radiotherapy stands as a cornerstone treatment for many solid tumors, primarily by damaging the DNA structure to inhibit tumor cell proliferation [127]. Radiotherapy uses high-energy ionizing radiation (IR) to disrupt DNA structure, inducing cell cycle arrest, senescence, and cell death [128, 129]. In addition, IR can induce indirect cellular effects through the radiolysis of cellular water and the stimulation of oxidases, thereby generating ROS that damage nucleic acids, proteins and lipids [128, 130]. One intriguing facet of IR is its capacity to induce the expression of ACSL4, resulting in elevated lipid peroxidation and ferroptosis in tumors [128]. Interestingly, as an adaptive response, IR also induces the expression of the ferroptosis inhibitors SLC7A11 and GPX4 [128]. Although ferroptosis is not directly associated with IR-induced DNA damage and repair, it plays a role in IR-induced tumor suppression. Notably, ferroptosis inducers sensitize radioresistant cancer cells to IR [128].

Telomerase is a key factor that regulates chromosome stability and integrity during DNA replication. BIBR1532, a highly selective telomerase inhibitor, has been shown to augment the radiosensitivity of non-small cell lung cancer by promoting IR-induced telomere dysfunction [131]. Furthermore, BIBR1532 can enhance IR-induced ROS and ferroptosis, inducing mitochondrial stress and the release of endogenous mitochondrial DNA into the cytoplasm [132]. This cascade activates the cGAS-STING pathway and then elicits an IFN-linked adaptive immune response, ultimately enhancing the anti-tumor efficacy of radioimmunotherapy [132].

Radiation-induced bystander effect (RIBE) is a term that describes how radiation causes oxidative stress and DNA damage not only in cancer cells directly targeted but also induces a damage response in neighboring unirradiated cancer cells or tissues [54]. RIBE is mediated mainly by soluble factors, including EVs or signaling molecules, released by irradiated cells [54]. For instance, Wan et al. reported that RIBE is predominantly mediated by irradiated tumor cell-released microparticles (RT-MPs) which can induce immunogenic ferroptosis, and polarize M2-TAMs to M1-TAMs, reversing the tumor-inhibitory TME [54]. Moreover, Deng et al. designed a therapeutic system utilizing RSL3- loaded radiated tumor cell-derived microparticles (RC@RMPs) to induce ferroptosis and remodel the TME in prostate cancer, highlighting the potential of exploiting RIBE for therapeutic benefit [133]. These data suggest the promising perspective of ferroptosis inducers combined with IR in treating tumors.

### Nanoparticle-based strategies to specifically induce ferroptosis in the TME

Although ferroptosis inducers such as erastin and RSL3 have shown promising anti-cancer effects in vitro, their limited in vivo bioactivities and potential side effects limit their clinical application. Owing to their ability to selectively target tumor cells and their unique physicochemical properties, nanoparticles emerge as promising candidates for cancer therapy [134]. Nanoparticles can also surmount biological barriers that impede traditional cancer therapies, such as poor solubility, rapid clearance, and limited penetration in solid tumors [134]. Various nanoparticle technologies, including micelles, liposomes, exosomes, polymersomes, polymeric nanoparticles, dendrimer nanoparticles, protein nanoparticles, inorganic nanoparticles, biomimetic nanoparticles, and hybrid nanoparticles, offer versatile platforms for drug delivery [134–137].

The immunosuppressive TME is a key factor that induces drug resistance. Classic approaches to reversing the immunosuppressive TME involve activating T cells, triggering DC maturation, or improving their antigen presentation. Ferroptosis induction appears to be a promising strategy to reprogram the TME and then sensitize other cancer treatments. Liu et al. reported a thermal-responsive SPH_GA_ that efficiently induces ferroptosis and immunogenic cell death, reversing the immunosuppressive TME and enhancing anti-tumor immune responses [138]. Wang et al. constructed a BSA-FA functionalized iron-containing metal-organic framework (TPL@TFBF) that efficiently trigger ferroptosis and pyroptosis in cancer cells, inhibiting melanoma growth and metastasis [139]. This nanoparticle also stimulates antigen presentation of DCs and the proliferation of cytotoxic T lymphocytes, enhancing PD-L1 blockade efficacy [139]. The cysteine/GSH/GPX4 mechanism is widely employed in ferroptosis-related nanoparticle cancer therapy research. For example, Ruan et al. developed an engineered microbial nanohybrid based on *Escherichia coli* and Cu_2_O nanoparticles (E. coli@Cu_2_O) that can induce ferroptosis by inactivating GPX4, thereby reversing the immunosuppressive TME and increasing PD-1 blockade efficacy [140]. Additionally, nanoparticles, including manganese molybdate nanoparticles (MnMoOx NPs) [141], Ca & Mn dual-ion hybrid nanostimulator (CMS) [142], and PDPCA@ATRA [143], have been reported to trigger ferroptosis, reverse the immunosuppressive TME, and enhance anti-tumor immunity by depleting GSH.

The low immunogenicity of the tumor itself poses a serious obstacle in effectively treating immunogenic “cold” tumors. Fan et al. designed a nanozyme, Fe-TCPP-R848-PEG (Fe-MOF-RP), which catalyzes the decomposition of H_2_O_2_ within the tumor, inducing tumor cell ferroptosis and immunogenic cell death [144]. This nanozyme also repolarizes M2 TAMs into M1 macrophages, reshaping the immunosuppressive TME [144]. Similarly, Wang et al. devised a self-assembled nanoparticle (TEP-FFG-CRApY) that induces ferroptosis and immunogenic cell death by degrading the GPX4 protein and promoting the Fenton reaction [145]. Liu et al. proposed nanosheets that reinforce ferroptosis though GSH depletion and hydroxyl radical generation [146]. Furthermore, ferroptosis-triggered immunogenic cell death and nanosheet-layered IFN-γ facilitate DC maturation and T cell priming, contributing to immunogenic ferroptosis of tumor cells [146]. Lei et al. synthesized manganese molybdate nanoparticles (MMP NDs) that consume GSH and high-valence Mo and Mn to induce ferroptosis, eliciting tumor-specific immune responses [147].

Iron can serve as a catalyst to control the Fenton reaction [20]. Iron-based ternary chalcogenide nanoparticles (AgFeS2) can release Fe^3+^ to effectively catalyze the Fenton reaction, resulting in ferroptosis and immunogenic cell death [148]. In addition, Zhang et al. designed a glucose oxidase (GOx)-loaded H_2_O_2_-responsive Fe_3_O_4_-based nanoparticle (Fe_3_O_4_@GOx@Tuftsin@Lipids) that enhances the uptake of Fe_3_O_4_ by tumor cells, triggering strong ferroptosis and infiltration of T lymphocytes in tumor tissues [149].

Furthermore, macrophage-based nanoparticles are emerging as a method to induce ferroptosis. For example, Feng et al. developed a strategy that utilizes iron-based nanoparticle-regulated TAMs to target cancer stem cell niches and trigger ferroptosis [150]. Wang et al. proposed a strategy involving the use of an M1 macrophage membrane-camouflaged ferrous-supply-regeneration nanoplatform (M1mDDTF) to synergistically reinforce immunogenic ferroptosis and reprogram TAMs [151]. Although many nanoparticles have been designed to specifically induce cancer cell ferroptosis in preclinical animal models, further studies and clinical trials should be performed to evaluate their application value.

## Conclusions and perspectives

Ferroptosis has drawn significant attention due to its crucial role in tumor inhibition and its promising potential as a novel strategy to combat therapy resistance. Preclinical investigations have confirmed that combining ferroptosis inducers with traditional cancer treatments exhibit synergistic anti-cancer effects. However, recent studies have revealed a new paradigm. In addition to facilitating cancer cell death, ferroptosis, under certain conditions, can induce potent immune suppression in the TME by affecting both innate and adaptive immune responses. Developing combination therapy methods that can induce ferroptosis and reverse immunosuppressive TME seems to be a potential strategy. In addition, potential adverse effects of ferroptosis activators have been observed in several cancer models, and specific ferroptosis inducers with high bioavailability and safety is still lacking.

Given the intricate roles of ferroptosis in both cancer cells and non-cancer cells in the TME, identifying the most susceptible cells to ferroptosis is crucial, and the development of cell-specific ferroptosis inducers or targeted drug delivery systems presents a challenging yet essential task. Some nanoparticle-based strategies have been developed to specifically induce ferroptosis in the TME. Of them, the natural nanoparticles EVs show promising prospect for their high biocompatibility, circulating stability, the ability to cross biological barriers, and minimal immunogenicity. Engineered EVs hold the potential to specifically delivery ferroptosis inducers into specific cell types in the TME, inhibitory tumor growth [10, 135, 136].

Furthermore, ferroptosis drugs usually exert tumor-inhibitory effect in a context-dependent manner, and screening suitable cancer patient for specific ferroptosis inducer is challenging. Consequently, identifying reliable biomarkers that can efficiently predict patient responses to ferroptotic therapy and potential adverse effects is essential for implementing precision therapy strategies.

In conclusion, to fully exploit the therapeutic potential of ferroptosis induction, comprehensive basic, preclinical and clinical investigations are imperative to address these complex issues and pave the way for optimized therapeutic strategies in the future.

## Data Availability

Not applicable.

## Abbreviations

AA: Arachidonic acid
Acod1: Aconitate decarboxylase 1
ACT: Adoptive cell therapy
ACSL4: Acyl-CoA synthetase long chain family member 4
ALOXs: Arachidonate lipoxygenases
ANO1: Anoctamin 1
BAP1: BRCA1-associated protein 1
BCAT2: Branched-chain amino acid aminotransferase 2
BET: Bromodomain and extra-terminal domain
CAD: Cytosolic dihydroorotase
CAFs: Cancer-associated fibroblasts
CoQ: Coenzyme Q10, also known as ubiquinone-10
CoQH_2_: Ubiquinol
CRC: Colorectal cancer
CyTOF: Cytometry by time of flight
DCs: Dendritic cells
DEPDC5: DEP domain containing 5
DHODH: Dihydroorotate dehydrogenase
EVs: Extracellular vesicles
FGF5: Fibroblast growth factor 5
FGFR2: Fibroblast growth factor receptor 2
FH: Fumarate hydratase
FSTL1: Follistatin like protein 1
FPS: Ferroptosis score
FSP1: Ferroptosis suppressor protein 1
FTL: Ferritin light chain
GOT1: Glutamate oxaloacetate transaminase 1
GOx: Glucose oxidase
GPX4: Glutathione peroxidase 4
GSH: Glutathione
HCC: Hepatocellular carcinoma
ICI: Immune checkpoint inhibitor
IFN: γ-Secreting interferon gamma
IL: 9-Interleukin 9
IR: Ionizing radiation
KEAP1: Kelch like ECH associated protein 1
KMT2B: Lysine methyltransferase 2B
LACTB: Lactamase beta
LCN2: Lipocalin-2
LPCAT3: Lysophosphatidylcholine acyltransferase 3
MAT1A: Methionine adenosyltransferase 1 A
MDSCs: Myeloid-derived suppressor cells
NK: Natural killer
NSCLC: Non-small cell lung cancer
OH: Hydroxyl radical
PE: Phosphatidylethanolamine
PGAM1: Phosphoglycerate mutase 1
PGE2: Prostaglandin E2
PLs: Phospholipids
PMNs: Pathologically activated neutrophils
PRMT5: Protein arginine methyltransferase 5
PUFAs: Polyunsaturated fatty acids
RCD: Regulated cell death
RFL: Relative ferroptosis level
RIBE: Radiation-induced bystander effect
ROS: Reactive oxygen species
RT: MPs-Irradiated tumor cell-released microparticles
SAPE: OOH-1-steaoryl-2-15-HpETE-sn-glycero-3-phosphatidylethanolamine
scRNA: Seq-Single-cell RNA sequencing
SLC7A11 (xCT): Solute carrier family 7 member 11
SLC3A2: Solute carrier family 3 member 2
System Xc: Cystine-glutamate antiporter
TAMs: Tumor-associated macrophages
TANs: Tumor-associated neutrophils
TILs: Tumor-infiltrating lymphocytes
TME: Tumor microenvironment
TNBC: Triple-negative breast cancer
Tregs: Regulatory T cells
TrxR: Thioredoxin reductase
UMPS: Uridine 5′-monophosphate synthase
VDAC2/VDAC3: Voltage dependent anion channel 2/3
